# Physical activity and fitness are associated with verbal memory, quality of life and depression among nursing home residents: preliminary data of a randomized controlled trial

**DOI:** 10.1186/s12877-018-0770-y

**Published:** 2018-03-27

**Authors:** Haritz Arrieta, Chloe Rezola-Pardo, Iñaki Echeverria, Miren Iturburu, Susana Maria Gil, Jose Javier Yanguas, Jon Irazusta, Ana Rodriguez-Larrad

**Affiliations:** 1 Department of Physiology, Faculty of Medicine and Nursing, University of the Basque Country (UPV/EHU), Barrio Sarriena s/n, E-48940 Leioa, Bizkaia Spain; 2 Matia Instituto Gerontológico Foundation, Camino de los Pinos 35, E-20018 Donostia-San Sebastian, Gipuzkoa Spain

**Keywords:** Physical activity, Exercise, Cognition, Quality of life, Depression, Older adults, Nursing home

## Abstract

**Background:**

Few studies have simultaneously examined changes in physical, cognitive and emotional performance throughout the aging process.

**Methods:**

Baseline data from an ongoing experimental randomized study were analyzed. Physical activity, handgrip, the Senior Fitness Test, Trail Making Test A, Rey Auditory-Verbal Learning Test, Quality of Life-Alzheimer’s Disease Scale (QoL-AD) and the Goldberg Depression Scale were used to assess study participants. Logistic regression models were applied. Trial registration: ACTRN12616001044415 (04/08/2016).

**Results:**

The study enrolled 114 participants with a mean age of 84.9 (standard deviation 6.9) years from ten different nursing homes. After adjusting for age, gender and education level, upper limb muscle strength was found to be associated with Rey Auditory-Verbal Learning Test [EXP(B): 1.16, 95% confidence interval (CI): 1.04–1.30] and QoL-AD [EXP(B): 1.18, 95% CI: 1.06–1.31]. Similarly, the number of steps taken per day was negatively associated with the risk of depression according to the Goldberg Depression Scale [EXP(B): 1.14, 95% CI: 1.000–1.003]. Additional analyses suggest that the factors associated with these variables are different according to the need for using an assistive device for walking. In those participants who used it, upper limb muscle strength remained associated with Rey Auditory-Verbal Learning Test [EXP(B): 1.21, 95% CI: 1.01–1.44] and QoL-AD tests [EXP(B): 1.19, 95% CI: 1.02–1.40]. In those individuals who did not need an assistive device for walking, lower limb muscle strength was associated with Rey Auditory-Verbal Learning Test [EXP(B): 1.35, 95% CI: 1.07–1.69], time spent in light physical activity was associated with QoL-AD test [EXP(B): 1.13, 95% CI: 1.00–1.02], and the number of steps walked per day was negatively associated with the risk of depression according to the Goldberg Depression Scale [EXP(B): 1.27, 95% CI: 1.000–1.004].

**Conclusions:**

Muscle strength and physical activity are factors positively associated with a better performance on the Rey Auditory-Verbal Learning Test, QoL-AD and Goldberg Depression Scale in older adults with mild to moderate cognitive impairment living in nursing homes. These associations appeared to differ according to the use of an assistive device for walking. Our findings support the need for the implementation of interventions directed to increase the strength and physical activity of individuals living in nursing homes to promote physical, cognitive and emotional benefits.

**Trial registration:**

ACTRN12616001044415 (04/08/2016).

## Background

Aging is a dynamic and progressive decline in physical and cognitive performance leading to the loss of overall function for the activities of daily living. Increasing evidence supports an interaction between physical and cognitive impairment within the cycle of decline associated with aging [1]. In other words, brain health is strongly linked to physical health, and physical performance is, to a large extent, thought to be cognitively mediated.

Moreover, physical activity and exercise, as beneficial lifestyle factors, may attenuate or prevent cognitive decline associated with aging [2–4]. Multiple studies have highlighted the beneficial effects of aerobic exercise [5, 6], resistance training [7] and physical activity [8, 9] on cognitive function in older adults, although the neurophysiologic mechanisms driving these effects are not well understood. Further, physical and cognitive function could be linked to health-related quality of life [10] (QoL) and affective conditions [11] in older adults. Previous works examining these relationships are largely restricted to people with cognitive impairments. Nevertheless, a longitudinal study performed in healthy older community-dwelling adults, found that greater levels of physical activity were independently associated with better long-term health-related QoL in a follow-up period of six years [12].

Despite the evidence supporting associations between physical, cognitive and affective aspects related to the aging process, few studies considered these conditions simultaneously. In addition, to our knowledge, no such studies have focused on older adults who live in nursing home settings, although this is one of the fastest-growing demographics worldwide [13]. Older adults living in nursing homes are characterized by old age, a high prevalence of multi-morbidity, functional impairment, severe cognitive deficits, depression, and very low physical activity [14]. However, there is a subgroup of residents that maintains the ability to walk and some of these residents even present wandering behavior [15].

Many residents of nursing homes require assistive walking devices to carry out the activities of daily living. The need to incorporate the upper limbs for getting up from a chair or for discharging the body weight while walking will affect their physical performance, specifically those features associated with muscle strength of the upper limbs. Therefore, it may be pertinent to think that, if associations between physical, cognitive and affective aspects exist, they could be conditioned by the need to use assistive devices for walking.

Further, although recent initiatives have aimed at improving the quality of care in nursing homes [16, 17], physical and social inactivity remain a concern in these institutions [18, 19]. Investigating the associations between physical, cognitive and affective aspects in older adults living in nursing homes may provide valuable insights for guiding clinical practice and consequently support nursing home management in evidence based decisions.

With this in mind, we sought to evaluate the associations between physical fitness and physical activity, and cognitive performance, QoL and depression risk in older adults living in long-term (LT) nursing homes. We hypothesized that better physical fitness and higher levels of physical activity might be independent factors for better cognitive performance, better QoL and lower risk of depression in older adults living in LT nursing homes. Secondarily, we examined whether these potential associations could differ for residents who require an assistive device for walking (for example crutches or canes).

## Methods

### Study design and participants

**Fig. 1:**
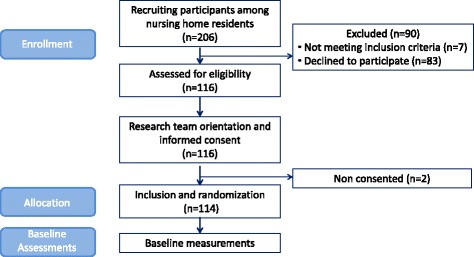
Flow diagram of the study

Data from a multicenter, randomized study carried out in ten LT nursing homes between October 2016 and June 2017 were available for analysis in this study. Seven residents out of 206 potential participants did not meet the inclusion criteria, 83 declined to participate, and two did not sign the informed consent document, leaving 114 participants. A flow diagram depicting the selection process is shown in Fig. 1. Details of the methods for designing and conducting the study were previously published [20]. Briefly, eligible participants included men and women aged ≥70 years, who scored ≥50 on the Barthel Index [21], scored ≥20 on the MEC-35 Test [22] (an adapted and validated version of the Mini Mental State Examination in Spanish), and who were able to stand and walk independently for at least ten meters. The study was approved by the Committee on Ethics in Research at the University of the Basque Country (Humans Committee Code M10/2016/105). The protocol is registered under the Australian and New Zealand Clinical Trials Registry (ANZCTR) with the identifier: ACTRN12616001044415. Date of registration: 04/08/2016.

### Measurements

**Physical activity** performed by the participants was objectively recorded with an accelerometer (Actigraph GT3X model, Actigraph LLC, Pensacola, FL, USA), worn on the hip with a belt for seven days. Activity was recorded using 60-s epochs. Data files recorded on the accelerometers were downloaded and processed with Actilife software (version 6, Actigraph, 2012). The analyzed variables were: number of steps per day and number of minutes per day spent in intensity specific categories. Selecting cut-off points to classify the intensity of physical activity in older people was difficult because there is no current consensus in the scientific literature. Thus, we followed the protocol developed by Freedson and collaborators [23], where the cut-off point for light physical activity was set in the range of 100–1951 counts per minute (cpm), and moderate to vigorous physical activity (MVPA) was defined as all activity ≥1952 cpm. The number of minutes per day at different intensities was calculated by summing all minutes where the count met the criterion for the specific intensity and then dividing by the number of valid days.

**Physical fitness** was assessed through the handgrip strength test [24] (Jamar dynamometer) of the dominant upper limb and the Senior Fitness Test [25] (SFT), a battery of six independent tests encompassing: chair stand test (lower limb strength), arm curl test (upper limb strength), six minute walking test (6MWT) (aerobic endurance), chair sit and reach test (lower limb flexibility), back scratch test (upper limb flexibility) and the 8-ft up and go test (dynamic balance).

**Cognitive performance tests** were evaluated by the same trained neuropsychologist; assessments were carried out individually in participants` own rooms. The MEC-35 test [22] was used for screening and scaling cognitive impairment. Trail Making Test A [26] was administered to assess the speed of information processing, and more concretely, aspects of motor control, motor speed and visual scanning speed. To administer the Trail Making Test A, participants were instructed to draw lines connecting consecutively numbered circles as quickly as possible. The resultant score is the number of seconds required to complete the task; shorter time indicates better performance. A Spanish validated version of the Rey Auditory-Verbal Learning Test [27] (RAVLT) was administered to participants to assess verbal memory. The test lasted approximately 15 min. Consisted of two lists that had to be read aloud; one of 15 words (List A) and a new list of 15 different words (List B). The participant was asked to freely recall the words read aloud by the evaluator in List A. Four more trials were performed in the same way. After five trials, the List B was presented, and a free recall trial was asked for the words in List B. Immediately after, participants were asked to freely recall again the words in List A. Twenty minutes later the participants were asked to recall the words on List A. Then, the evaluator read aloud the 30 words from List A and List B, and the participants were asked to recognize the words from List A. Even though RALVT can be analyzed trial by trial, the authors recommend to establish different measures for its clinical use [28]. In this study, the Total Learning measure (RAVLT-AT) was calculated, which evaluates the capacity to recall and to accumulate words through the 5 learning trials. The RAVLT-AT score resulted from the sum of the five consecutive learning trials (trial1 + trial2 + trial3 + trial4 + trial5).

**Health-related quality of life** was evaluated by a Spanish validated version of the Quality of Life-Alzheimer’s Disease Scale [29] (QoL-AD). Considering that many of the participants showed different levels of cognitive impairments or were at risk to develop dementia during the program, we selected the QoL-AD scale as the best tool for assessing health-related quality of life in our participants. The scale comprises 13 items (physical health, energy, mood, living situation, memory, family, marriage, friends, self as a whole, ability to do chores, ability to do things for fun, money and life as a whole). Each item is answered according to a Likert scale from 1(poor) to 4 (excellent), for a total score between 13 and 52, with higher scores indicating better QoL.

**Depression** was measured by the Goldberg Depression Scale [30] (GDS), which comprises four screening items and five supplementary ones. Participants who respond positively to two or more screening items go on responding to the following five. Participants scoring two or more have a 50% chance of having a clinically important disturbance of depression.

### Statistical analysis

Continuous variables were expressed as means with standard deviations (SD), and categorical variables as frequency counts and percentages (%). Taking into account that up-to-date reference values for the dependent variables MEC-35, Trail Making A, RAVLT-AT, and QoL-AD have not been reported for older adults living in nursing homes, the cut-off point in the current study was determined according to the median, as used in other studies [31, 32]. Thus, the dependent continuous variables were transformed into binary variables according to whether they had a value above or below their median. Comparisons of sociodemographic characteristics, physical fitness and physical activity between participants who were above or below the median on MEC-35, Trail Making A, RAVLT-AT, QoL-AD and Goldberg Depression Scale were performed using appropriate statistical tests according to the type and distribution of the data: *t-*test or Mann-Whitney *U*-test for continuous variables and Chi-squared test for categorical variables. A *p* value < 0.05 was considered significant. We also performed logistic regressions, with demographic, accelerometry and physical fitness data as independent variables, and cognitive performance tests, health-related quality of life and depression risk data as dependent variables. Those variables that reached a *p* value < 0.05 on univariate analysis were considered eligible for entry into the multiple logistic regression analysis. Backward regression models were then fitted. All multiple models were adjusted for age and gender. In addition, Hosmer-Lemershow goodness-of-fit, Omnibus and Nagelkerke’s R^2^ values for each model were specified. A Hosmer-Lemershow test was used to determine the goodness-of-fit of the models, that is, to determine if the observed event rates matched expected ones; a number closest to 1 show a better goodness of fit. Omnibus was used to test whether the explained variance was significantly greater than the unexplained variance; a *p* value < 0.05 was considered significant. Nagelkerke’s R^2^ values estimated the proportion of the dependent variable explained by the independent variables. Then, the sample was divided according to the need of any assistive device for walking, and multiple regression models were performed for each group. Statistical analysis was performed using SPSS v.21 software.

## Results

### Characteristics of study participants

This study adheres to the Consolidated Standards of Reporting Trials (CONSORT) guidelines. The study included 114 participants from ten LT nursing homes. Accelerometer monitoring showed that they performed very low levels of physical activity during the day (Table 1). Further, 46% of the participants scored lower than the median level of QoL-AD, and 25% of the participants had a 50% chance of having a clinically important disturbance of depression according to the Goldberg depression scale. In addition, 55% of the participants needed an assistive device for carrying out the activities of daily living.

**Table 1:** Baseline characteristics of the participants (*n* = 114)

Variable	Value
Age (yr) mean (SD)	84.9 (6.9)
70–79 (yr) %(n)	22.8% (26)
80–89 (yr) %(n)	53.5% (61)
≥ 90 (yr) %(n)	23.7% (27)
Sex	
Men %(n)	28.9% (33)
Women %(n)	71.1% (81)
Barthel Index mean (SD)	80.6 (12.9)
MEC-35 mean (SD)	27.5 (3.8)
BMI (kg/m^2^) mean (SD)	28.2 (5.1)
Education	
≤ 12 years %(n)	92% (104)
> 12 years %(n)	8% (10)
Steps/day mean (SD)	1162.3 (1776.4)
Light physical activity (min/day) mean (SD)	86.7 (59.6)
MVPA (min/day) mean (SD)	1.3 (3.1)
Handgrip (Kg) mean (SD)	21.1 (8.1)
Chair stand test (rep) mean (SD)	7.6 (3.9)
Arm curl test (rep) mean (SD)	12.2 (4.3)
6-min walk test (m) mean (SD)	230.7 (95.9)
Chair sit-and-reach test (cm) mean (SD)	−11.8 (9.3)
Back scratch test (cm) mean (SD)	−21.3 (12.9)
8-ft up-and-go test (sec) mean (SD)	15.4 (9.3)

*Abbreviations*: *MEC-35* Mini Examen Cognoscitivo-35, *BMI* Body Mass Index, *MVPA* moderate-vigorous physical activity, *rep* repetitions

### Participant characteristics according to their performance on MEC-35 test, trail making a test, RAVLT-AT test, QoL-AD test and Goldberg depression scale

Individuals who scored below the median of the sample on the MEC-35 test presented less flexibility of the lower limbs (*p* = 0.011) compared to the participants scoring equal to or higher than the median of the sample (Table 2). Similarly, participants who scored below the median of the sample on the RAVLT-AT test presented lower muscle strength in both upper and lower limbs (Chair stand test *p* = 0.009; Arm curl test *p* = 0.005) along with lower flexibility in the chair sit-and-reach test (*p* = 0.015) than individuals scoring equal to or higher than the median of the sample. In addition, those perceiving their QoL below the median of the sample presented lower levels of physical activity (Steps/day *p* = 0.007; Light physical activity *p* = 0.013) and lower levels of muscle strength (Handgrip test *p* = 0.032; Chair stand test *p* = 0.025; Arm curl test *p* = 0.002) compared to those perceiving their QoL equal to or higher than the median (Table 3). Older adults with a 50% chance of having a clinically relevant disturbance of depression according to the Goldberg depression scale scored lower in terms of physical activity (Steps/day *p* = 0.004; Light physical activity *p* = 0.009) and lower body muscle strength (Chair stand test *p* = 0.048; Arm curl test *p* = 0.004) than older adults with no risk of depression. There were no significant differences in physical fitness or physical activity characteristics of the participants in this study between those individuals above and below the median in Trail Making A test performance.

**Table 2:** Characteristics of the participants according to their MEC-35 test, Trail Making A test and RAVLT-AT test performance

	MEC-35		Trail Making A		RAVLT-AT	
Variables	< median	≥ median	< median	≥ median	< median	≥ median
Age (yr) mean (SD)	85.3 (8.0)	84.5 (5.8)	84.6 (6.2)	84.6 (5.6)	85.1 (5.8)	84.5 (7.4)
Sex						
Men %	29.4%	29.0%	34.4%	31.2%	36.2%	25.5%
Women %	70.6%	71.0%	65.6%	68.8%	63.8%	74.5%
Barthel Index mean (SD)	79.8 (12.5)	81.8 (13.4)	81.7 (12.8)	80.6 (14.2)	82.6 (11.7)	80.7 (12.9)
BMI (kg/m^2^) mean (SD)	28.6 (5.5)	27.8 (4.8)	28.0 (5.3)	28.0 (5.1)	27.4 (4.6)	28.8 (5.6)
Education						
≤ 12 years %	96.1%	88.5%	100%	81.2%^*^	89.1%	92.2%
> 12 years %	3.9%	11.5%	–	18.8%	10.9%	7.8%
Steps/day mean (SD)	912.3 (1326.1)	1248.5 (1967.1)	1376.0 (2575.2)	1312.4 (1649.8)	888.7 (702.8)	1375.2 (2363.9)
Light physical activity (min/day) mean (SD)	79.1 (59.5)	85.4 (58.7)	95.6 (69.7)	89.9 (59.8)	82.7 (52.6)	90.3 (65.9)
MVPA (min/day) mean (SD)	0.7 (0.6)	1.8 (4.2)	1.9 (5.2)	0.6 (0.6)	0.9 (1.0)	1.7 (4.5)
Handgrip (Kg) mean (SD)	19.9 (6.9)	21.8 (8.6)	22.3 (8.9)	21.8 (8.2)	20.9 (7.0)	21.2 (7.5)
Chair stand test (rep) mean (SD)	7.2 (3.4)	7.9 (4.5)	8.3 (4.0)	7.4 (4.3)	6.5 (3.7)	8.8 (3.9)^**^
Arm curl test (rep) mean (SD)	11.3 (3.7)	12.9 (4.5)	12.6 (4.4)	12.7 (3.7)	11.0 (3.8)	13.4 (4.1)^**^
6-min walk test (m) mean (SD)	220.9 (96.7)	241.8 (92.7)	253.5 (85.9)	233.1 (93.1)	234.6 (91.6)	231.2 (90.4)
Chair sit-and-reach test (cm) mean (SD)	− 13.4 (8.8)	− 9.8 (9.4)^*^	− 11.7 (8.9)	− 10.8 (9.9)	− 13.6 (8.5)	− 9.8 (10.1)^*^
Back scratch test (cm) mean (SD)	− 23.5 (13.1)	− 18.4 (12.8)	− 23.1 (14.0)	− 16.5 (13.1)	− 21.9 (13.8)	− 19.7 (12.7)
8-ft up-and-go test (sec) mean (SD)	16.2 (8.8)	14.4 (9.5)	13.4 (6.5)	14.4 (7.0)	14.9 (6.7)	14.5 (8.3)

*Abbreviations*: *MEC-35* Mini Examen Cognoscitivo-35, *RAVLT-AT* Rey Auditory-Verbal Learning Test-Total Learning, *BMI* Body Mass Index, *MVPA* moderate-vigorous physical activity, *rep* repetitions

^*^ *p* < 0.05

^**^ *p* < 0.01

**Table 3:** Characteristics of the participants according to their performance on the QoL-AD Test and their depression risk assessed by the Goldberg Depression Scale

	QoL-AD Test		Goldberg Depression Scale	
Variables	< median	≥ median	50% risk Depression	No depression risk
Age (yr) mean (SD)	85.4 (6.4)	84.0 (7.2)	86.1 (6.7)	84.2 (6.9)
Sex				
Men %	25.5%	34.5%	11.5%	36.8%^*^
Women %	74.5%	65.5%	88.5%	63.2%
Barthel Index mean (SD)	81.1 (12.9)	81.2 (12.6)	80.8 (14.4)	81.2 (12.1)
BMI (kg/m^2^) mean (SD)	27.7 (4.7)	28.7 (5.6)	28.7 (5.5)	28.1 (5.1)
Education				
≤ 12 years %	93.5%	89.1%	96.2%	89.3%
> 12 years %	6.5%	10.9%	3.8%	10.7%
Steps/day mean (SD)	742.40 (727.20)	1484.2 (2266.8)^**^	554.7 (295.3)	1339.7 (1997.2)^**^
Light physical activity (min/day) mean (SD)	70.84 (44.61)	99.8 (67.0)^*^	61.7 (37.5)	94.8 (63.0)^**^
MVPA (min/day) mean (SD)	0.9 (1.7)	1.6 (4.2)	0.9 (1.2)	1.5 (3.7)
Handgrip (Kg) mean (SD)	19.38 (6.91)	22.7 (8.0)^*^	18.7 (5.4)	22.0 (8.2)
Chair stand test (rep) mean (SD)	6.80 (3.96)	8.4 (3.9)^*^	6.7 (3.9)	7.9 (4.0)^*^
Arm curl test (rep) mean (SD)	10.98 (4.19)	13.4 (3.7)^**^	10.4 (3.0)	12.9 (4.2)^**^
6-min walk test (m) mean (SD)	219.92 (75.90)	242.3 (100.8)	221.8 (72.5)	235.4 (95.8)
Chair sit-and-reach test (cm) mean (SD)	− 11.56 (9.29)	− 11.5 (9.5)	− 12.9 (8.7)	− 11.1 (9.6)
Back scratch test (cm) mean (SD)	− 21.22 (15.12)	− 20.9 (11.7)	− 20.2 (15.1)	− 21.2 (12.8)
8-ft up-and-go test (sec) mean (SD)	16.34 (8.88)	13.41 (6.2)	15.5 (7.3)	14.5 (7.8)

*Abbreviations*: *QoL-AD Test* Quality of Life-Alzheimer Disease Test, *BMI* Body Mass Index, *MVPA* moderate-vigorous physical activity, *rep* repetitions

^*^ *p* < 0.05

^**^ *p* < 0.01

### Logistic regression models

We applied univariate logistic regression models to determine associations between each dependent and independent variable (Appendices 1, 2, 3, 4, 5 and 6). Those independent variables that reached a *p* value < 0.05 on the univariate analysis were included in the multiple logistic regression models that are detailed below (Tables 4, 5, 6 and 7).

**Table 4:** Logistic regression models according to the RAVLT-AT performance adjusted by age, sex, and education level

	Whole sample		
	B	EXP(B) (95% CI)	*p*-value
Arm curl test	0.15	1.16 (1.04–1.30)	0.009
Chair sit-and-reach test	0.04	1.05 (1.00–1.10)	0.039
Variables in the model: chair stand test, arm curl test and chair sit-and-reach test; Estimates are based on *n* = 95 participants due to missing values; Hosmer-Lemershow goodness of fit, *p* = 0.444; Omnibus, *p* = 0.001; R^2^ Nagelkerke = 0.175.			
	No aids for walking (*n* = 46)		
	B	EXP(B) (95% CI)	*p*-value
Chair stand test	0.29	1.35 (1.07–1.69)	0.010
Hosmer-Lemershow goodness of fit, *p* = 0.811; Omnibus, *p* = 0.004; R^2^ Nagelkerke = 0.225.			
	Aids for walking (*n* = 49)		
	B	EXP(B) (95% CI)	*p*-value
Arm curl test	0.19	1.21 (1.01–1.44)	0.035
Chair sit-and-reach test	0.07	1.08 (1.00–1.16)	0.040
Hosmer-Lemershow goodness of fit, *p* = 0.405; Omnibus, *p* = 0.008; R^2^ Nagelkerke = 0.285.

**Table 5:** Logistic regression models according to the QoL-AD performance adjusted by age and sex

	Whole sample		
	B	EXP(B) (95% CI)	*p*-value
Arm curl test	0.17	1.18 (1.06–1.31)	0.003
Variables in the model: steps/day, light physical activity, handgrip, chair stand test, arm curl test; Estimates are based on *n* = 99 participants due to missing values; Hosmer-Lemershow goodness of fit, *p* = 0.905; Omnibus, *p* = 0.001; R^2^ Nagelkerke = 0.132.			
	No aids for walking (*n* = 45)		
	B	EXP(B) (95% CI)	*p*-value
Light physical activity*	0.01	1.132 (1.00–1.02)	0.048
* One unit = 10 min of light physical activity/day Hosmer-Lemershow goodness of fit, *p* = 0.911; Omnibus, *p* = 0.020; R^2^ Nagelkerke = 0.152.			
	Aids for walking (*n* = 54)		
	B	EXP(B) (95% CI)	*p*-value
Arm curl test	0.18	1.19 (1.02–1.40)	0.026
Hosmer-Lemershow goodness of fit, *p* = 0.545; Omnibus, *p* = 0.016; R^2^ Nagelkerke = 0.136.

**Table 6:** Logistic regression models according to the Goldberg Depression Scale adjusted by age, sex and education level

	Whole sample		
	B	EXP(B) (95% CI)	*p*-value
Steps/day*	0.001	1.142 (1.000–1.003)	0.028
* One unit = 100 steps/day Variables in the model: steps/day, light physical activity, arm curl test; Estimates are based on n = 99 participants due to missing values; Hosmer-Lemershow goodness of fit, *p* = 0.031; Omnibus, *p* = 0.000; R^2^ Nagelkerke = 0.209.			
	No aids for walking (n = 45)		
	B	EXP(B) (95% CI)	*p*-value
Steps/day*	0.002	1.274 (1.000–1.004)	0.022
* One unit = 100 steps/day Hosmer-Lemershow goodness of fit, *p* = 0.326; Omnibus, *p* = 0.001; R^2^ Nagelkerke = 0.419.			
	Aids for walking (n = 54)		
	B	EXP(B) (95% CI)	*p*-value
Sex (Female)	−2.169	0.114 (0.014–0.961)	0.046
Hosmer-Lemershow goodness of fit, *p* = .; Omnibus, *p* = 0.012; R^2^ Nagelkerke = 0.159.

**Table 7:** Logistic regression models according to the MEC-35 performance adjusted by age, sex and education level

	Whole sample		
	B	EXP(B) (95% CI)	*p*-value
Chair sit-and-reach test	0.06	1.06 (1.02–1.11)	0.006
Estimates are based on *n* = 104 participants due to missing values; Hosmer-Lemershow goodness of fit, *p* = 0.587; Omnibus, *p* = 0.004; R^2^ Nagelkerke = 0.13.			
	No aids for walking (n = 49)		
	B	EXP(B) (95% CI)	*p*-value
Chair sit-and-reach test	0.06	1.07 (0.99–1.14)	0.053
Hosmer-Lemershow goodness of fit, *p* = 0.138; Omnibus, *p* = 0.042; R^2^ Nagelkerke = 0.108.			
	Aids for walking (*n* = 55)		
	B	EXP(B) (95% CI)	*p*-value
Chair sit-and-reach test	0.05	1.05 (0.99–1.11)	0.09
Hosmer-Lemershow goodness of fit, *p* = 0.103; Omnibus, *p* = 0.083; R^2^ Nagelkerke = 0.071.			

### Factors associated with RAVLT-AT test performance

After adjusting for age, gender and education level, the variables that were associated with a RAVLT-AT test score equal to or above the median of the sample were upper limb muscle strength and lower limb flexibility (Table 4). To address the second objective of the study, we divided the sample according to the need of any assistive device for walking and performed the same regression model in each group. After adjusting for age, gender and education level, the lower limb muscle strength was associated with a score in the RAVLT-AT test equal to or above the median in those individuals who did not need any assistive device for walking. Upper limb muscle strength and flexibility in the chair sit-and-reach test were associated with a score equal to or higher than the median on the RAVLT-AT test in those individuals needing any assistive device for walking.

### Factors associated with QoL-AD test performance

Regarding QoL, the multiple regression model performed with the whole sample revealed that upper limb muscle strength was associated with a score equal to or higher than the median on the QoL-AD test (Table 5). When we stratified the sample according to the use of any assistive device for walking, light physical activity was associated with a QoL-AD score equal to or higher than the median in those individuals who did not need any help for walking, while upper limb muscle strength appeared to be associated for those needing assistance.

### Factors associated with Goldberg depression scale performance

In the regression model performed with the whole sample, the number of steps/day walked by the participants was associated with the absence of risk of depression according to the Goldberg Depression Scale (Table 6). In those individuals who did not need any assistive device for walking, the number of steps/day was again associated with the absence of risk of depression. In addition, in those individuals needing walking assistance, female gender was associated with a 50% greater risk of depression.

### Factors associated with MEC-35 test performance

Finally, when the whole sample was analyzed, chair sit and reach test was associated with performance on the MEC-35 test equal to or above the median of the sample (Table 7). No independent variables were found to be associated with higher score on MEC-35 performance when the analysis was stratified according to the use of an assistive device for walking.

## Discussion

The results of this study showed that physical fitness and, more specifically, upper limb muscle strength were associated with RAVLT-AT and QoL-AD tests in older adults living in LT nursing homes. Similarly, the number of steps taken by the participants per day was negatively associated with the risk of depression according to the Goldberg Depression Scale. Lower limb flexibility was also associated with a better score on the MEC-35 test. Additional analyses suggest that the factors associated with these variables are different according to the need for using an assistive device for walking. In those participants who used an assistive device for walking, upper limb muscle strength remained associated with RAVLT-AT and QoL-AD tests. In those individuals who did not need any assistive device for walking, lower limb muscle strength was associated with RAVLT-AT test, the time spent in light physical activity proved to be associated with QoL-AD test, and the number of steps walked by the participants remained a factor negatively associated with the risk of depression according to the Goldberg Depression Scale.

The results of the current study partially support our hypothesis that better physical fitness and higher levels of physical activity might be factors associated with better performance in the RAVLT-AT test, the QoL-AD test, the MEC-35 test or the Goldberg Depression Scale. However, we found that specific parameters of physical fitness (muscle strength and the level of physical activity in particular) were associated with specific cognitive variables. Other studies have recently observed this specificity in the link between physical and cognitive performance in the older adult population. An intervention study [33] reported a dose-response effect of aerobic exercise on components of visuospatial function in a group of community-living older sedentary adults without cognitive impairment. Another prospective study [34] found a dose-response effect of resistance training on executive cognitive function of selective attention and conflict resolution among senior community-dwelling women aged 65 to 75 years. In addition, links between physical activity and processing speed have also been observed [3, 35, 36]. Nevertheless, to our knowledge, no study has assessed the specificity in the association between physical, cognitive and emotional functions among LT nursing home residents. Previous works have focused exclusively on high functioning older community-dwelling adults or have been largely restricted to people with cognitive impairments [37, 38]. Thus, this is the first study identifying muscle strength and physical activity as factors that could explain a better verbal memory, better QoL and lower risk of depression in older adults living in LT nursing homes.

The regression model showed that for a one-unit increase in the arm curl test (one repetition), the probability of performing at or above the median on the RAVLT-AT test increased by 16%. This is a novel finding of the potential mediating effects of muscle strength on the verbal memory capacity of the participants. This result is in agreement with other studies that have identified strength as a factor mediating cognitive adaptations in older adults [38–40]. Yet, data on the effects of resistance-based exercise programs on cognitive parameters are scarce. Including a combination of multiple exercise modalities, particularly resistance training, in long-term exercise programs is reported to enhance cognition in the older population to a greater extent than programs including only aerobic training [3]. In addition, the evidence concerning the possible association of muscle strength with QoL is more limited. Further, a one-unit increase in the arm curl test (one repetition) also led to a higher probability of performing at or above the median on the QoL-AD test by 18%. Thus, the current study provides new data on the potential associations between muscle strength and RAVLT-AT and QoL-AD tests that warrant further investigation. It could be hypothesized that encouraging older adult living in LT nursing homes to engage in exercise programs that include resistance training could benefit not only physical but also cognitive function.

In addition, for an increase of 100 steps/day in the physical activity of the participants, the probability of being in the group with no risk of depression according to the Goldberg Depression Scale increased by 14%. Hence, physical activity could be proposed as a protective factor for reducing the risk of suffering from depression. This result aligns with other studies finding that depression in older people living in nursing homes is correlated, among other factors, with the activities performed outside the nursing home [41]. Thus, the higher their level of physical activity, the more opportunities could arise for residents to visit personally meaningful places and to interact socially with others. In fact, the objectively measured physical activity of the participants was extremely low, which is consistent with previous studies reporting that nursing homes residents` life-space (that is, the spatial extension of an individual’s environment that s/he moves in during a specified time period [42]) is severely limited to private rooms and adjacent living units [43]. Thus, nowadays, there is sufficient evidence to support the urgent implementation of interventions aimed at encouraging physical activity of older adults living in nursing homes.

For a one-unit increase in the chair sit-and-reach test (one cm), the probability of performing at or above the median on the MEC-35 test increased by 6%. This unexpected finding in the association between flexibility and MEC-35 could be masking the difficulty patients have to understand the chair sit-and-reach test that we have observed during the assessments. Thus, it should be interpreted cautiously.

Our results also showed that the associations between the muscle strength and RAVLT-AT and QoL-AD tests are different according to the use of an assistive device for walking. In those participants needing assistance, the regression models demonstrated that a one-unit increase in the arm curl test (one repetition) increased the probability of performing at or above the median on the RAVLT-AT test by 21%, and on the QoL-AD test by 19%. Thereby, the association between upper limb strength and RAVLT-AT test performance is higher than that found when the whole sample was analyzed (from 16% to 21%). In contrast, in those participants who did not need any assistive device for walking, lower limb muscle strength was the variable associated with RAVLT-AT test, and time performing light physical activity was the variable associated with QoL-AD test. Specifically, for a one-unit increase in the chair stand test (one repetition), the probability of performing at or above than the median on the RAVLT-AT test increased by 35%. Further, for a 10-min/day increase in light physical activity, the probability of being in the group with a QoL-AD test score equal to or higher than the median increased by 13%. We can only speculate regarding these findings, but it could be related to how the participants used their upper or lower limbs to carry out the activities of daily life. For example, those older adults who need to incorporate the upper limbs for walking, for maintaining balance or for getting up from a chair may have undergone adaptations in the muscle physiology that could somehow influence the associations. Thereby, we surmise that participants with higher levels of well-being also have a more active lifestyle, and this could explain why they might have higher strength (this assumption could also work in the inverse sense). However, an alternative explanation could be that those individuals with a more active lifestyle could have higher strength and, consequently, might have higher levels of well-being (and vice versa).

According to the Goldberg Depression Scale and as seen for the whole cohort, the regression model in those participants that did not need aids for walking showed that for a 100-step/day increase in physical activity, the probability of being in the group with no risk of depression increased by 27%. In those participants who needed aids for walking, the regression model result showed that being female increased the probability of being in the group with 50% risk of depression, according to the Goldberg Depression Scale, by 11%. This result agrees with other studies where gender, specifically being female, has been identified as a risk factor for experiencing depression [44]. Nevertheless, an important limitation in this study when studying depression is the failure to consider other variables such as social support, comorbidity or pharmacology. The current study aimed at focusing only on the associations between physical conditions and depression risk, thus, these results should be interpreted cautiously.

Several molecular and physiological mechanisms have been proposed to link strength and cognition, including insulin-like growth factor, brain-derived neurotrophic factor, myokines, fibroblast growth factor 2, and vascular endothelial growth factor [7, 45, 46]. These factors are thought to enhance neurogenesis and to play a key role in the positive effects of exercise on cognition, although the mechanisms need to be fully investigated.

There are a few limitations to this study; first, it is limited by its cross-sectional nature, precluding any ability to ascertain temporality. Second, some variables that could also be relevant, such as social support, comorbidity or pharmacology, have not been assessed and thus the results should be interpreted with caution. Third, the results cannot be directly applied to all the nursing home residents; we could not ascertain whether these results would apply to those who refused participation or did not fulfill the physical and cognitive criteria. Finally, the strength of this study is that physical activity has been objectively measured through accelerometers and that the sample size is one of the largest among studies focused on the associations between physical, cognitive and emotional aspects of the aging processes that characterize nursing home residents.

## Conclusions

The present work described the associations between physical, cognitive and emotional performance in a sample of older adults living in LT nursing homes. Specifically, muscle strength and physical activity were factors associated with a better performance on the RAVLT-AT, QoL-AD and Goldberg Depression Scale. These associations appeared to differ according to the use of an assistive device for walking. Further investigation is required to understand the physiological mechanisms underlying links between skeletal muscle physiology, cognition and well-being in this vulnerable population. The results offer further evidence to support the urgent need to implement interventions directed to increase the strength and physical activity of individuals living in nursing homes, as they might benefit not only physically, but also in terms of cognitive and emotional functioning.

## Appendix 1

**Table 8:** Univariate logistic regression analysis with MEC-35 as dependent variable

	B	EXP(B) (95% CI)	*p*-value
Age	−0.017	0.983 (0.931–1.038)	0.539
Gender (Female)	0.018	1.019 (0.451–2.300)	0.965
Barthel Index	0.012	1.012 (0.984–1.042)	0.402
BMI	−0.033	0.968 (0.898–1.042)	0.382
Education (> 12 years)	1.156	3.176 (0.630–16.022)	0.162
Steps/day	0.000	1.000 (1.000–1.000)	0.336
Light physical activity	0.002	1.002 (0.995–1.008)	0.579
MVPA	0.362	1.436 (0.899–2.291)	0.130
Handgrip	0.032	1.033 (0.984–1.085)	0.196
Chair stand test	−0.004	0.996 (0.914–1.086)	0.933
Arm curl test	0.077	1.080 (0.984–1.184)	0.106
6-min walk test	0.002	1.002 (0.998–1.006)	0.362
Chair sit-and-reach test	0.053	1.055 (1.011–1.100)	0.014^*^
Backscratch test	0.030	1.031 (0.999–1.064)	0.059
8-ft up-and-go test	−0.009	0.991 (0.953–1.030)	0.644

Notes: with the exception of gender and education, all the independent variables are continuous

*Abbreviations*: *BMI* Body Mass Index, *MVPA* moderate-vigorous physical activity

^*^ *p* < 0.05

## Appendix 2

**Table 9:** Univariate logistic regression analysis with Trail Making-A test as dependent variable

	B	EXP(B) (95% CI)	*p*-value
Age	0.000	1.000 (0.919–1.088	0.997
Gender (Female)	1.142	1.152 (0.405–3.275)	0.790
Barthel Index	−0.007	0.993 (0.957–1.031)	0.728
BMI	0.000	1.000 (0.909–1.101)	0.997
Education (> 12 years)	21.379	1,926,143,082	0.999
Steps/day	0.000	1.000 (1.000–1.000)	0.906
Light physical activity	−0.001	0.999 (0.991–1.006)	0.727
MVPA	−0.389	0.678 (0.297–1.547)	0.356
Handgrip	−0.011	0.989 (0.932–1.049)	0.718
Chair stand test	−0.062	0.940 (0.837–1.054)	0.289
Arm curl test	0.020	1.020 (0.906–1.149)	0.740
6-min walk test	−0.004	0.996 (0.991–1.002)	0.206
Chair sit-and-reach test	0.022	1.022 (0.971–1.076)	0.395
Backscratch test	0.038	1.039 (0.997–1.083)	0.072
8-ft up-and-go test	0.045	1.046 (0.973–1.125)	0.226

Notes: with the exception of gender and education, all the independent variables are continuous

*Abbreviations*: *BMI* Body Mass Index, *MVPA* moderate-vigorous physical activity

## Appendix 3

**Table 10:** Univariate logistic regression analysis with RAVLT-AT test as dependent variable

	B	EXP(B) (95% CI)	*p*-value
Age	−0.023	0.978 (0.921–1.038)	0.460
Gender (Female)	0.505	1.656 (0.696–3.939)	0.254
Barthel Index	−0.021	0.980 (0.949–1.011)	0.207
BMI	0.032	1.032 (0.955–1.116)	0.425
Education (> 12 years)	−0.360	0.698 (0.176–2.774)	0.609
Steps/day	0.000	1.000 (1.000–1.001)	0.237
Light physical activity	0.002	1.002 (0.995–1.009)	0.532
MVPA	0.098	1.103 (0.917–1.326)	0.296
Handgrip	0.004	1.004 (0.950–1.060)	0.892
Chair stand test	0.133	1.142 (1.030–1.266)	0.012^*^
Arm curl test	0.148	1.159 (1.041–1.291)	0.007^**^
6-min walk test	0.000	1.000 (0.995–1.004)	0.847
Chair sit-and-reach test	0.053	1.054 (1.009–1.101)	0.018^*^
Backscratch test	0.013	1.013 (0.982–1.045)	0.421
8-ft up-and-go test	−0.009	0.991 (0.942–1.044)	0.743

Notes: with the exception of gender and education, all the independent variables are continuous

*Abbreviations*: *BMI* Body Mass Index, *MVPA* moderate-vigorous physical activity

^*^ *p* < 0.05

^**^ *p* < 0.01

## Appendix 4

**Table 11:** Univariate logistic regression analysis with QoL-AD test as dependent variable

	B	EXP(B) (95% CI)	*p*-value
Age	−0.030	0.970 (0.916–1.028)	0.309
Gender (Female)	−0.431	0.650 (0.275–1.534)	0.325
Barthel Index	0.000	1.000 (0.970–1.032)	0.975
BMI	0.036	1.036 (0.960–1.119)	0.359
Education (> 12 years)	0.563	1.755 (0..414–7.446)	0.446
Steps/day	0.001	1.001 (1.000–1.001)	0.039^*^
Light physical activity	0.010	1.010 (1.002–1.019)	0.019^*^
MVPA	0.078	1.082 (0.916–1.276)	0.354
Handgrip	0.061	1.063 (1.005–1.125)	0.034^*^
Chair stand test	0.110	1.116 (1.012–1.231)	0.028^*^
Arm curl test	0.166	1.181 (1.059–1.317)	0.003^**^
6-min walk test	0.003	1.003 (0.998–1.007)	0.231
Chair sit-and-reach test	0.012	1.012 (0.972–1.054)	0.558
Backscratch test	0.002	1.002 (0.972–1.033)	0.889
8-ft up-and-go test	−0.050	0.951 (0.902–1.003)	0.067

Notes: with the exception of gender and education, all the independent variables are continuous

*Abbreviations*: *BMI* Body Mass Index, *MVPA* moderate-vigorous physical activity

^*^ *p* < 0.05

^**^ *p* < 0.01

## Appendix 5

**Table 12:** Univariate logistic regression analysis with Goldberg Depression Scale as dependent variable

	B	EXP(B) (95% CI)	*p*-value
Age	−0.043	0.958 (0.895–1.025)	0.216
Gender (Female)	−1.498	0.224 (0.062–0.812)	0.023^*^
Barthel Index	0.002	1.002 (0.968–1.038)	0.895
BMI	−0.024	0.976 (0.896–1.063)	0.579
Education (> 12 years)	1.094	2.985 (0.355–25.094)	0.314
Steps/day	0.002	1.002 (1.000–1.003)	0.008^**^
Light physical activity	0.014	1.014 (1.003–1.026)	0.014^*^
MVPA	0.098	1.103 (0.852–1.428)	0.456
Handgrip	0.067	1.070 (0.997–1.148)	0.061
Chair stand test	0.108	1.114 (0.999–1.243)	0.051
Arm curl test	0.177	1.194 (1.052–1.356)	0.006^**^
6-min walk test	0.002	1.002 (0.997–1.007)	0.431
Chair sit-and-reach test	0.033	1.033 (0.984–1.084)	0.188
Backscratch test	−0.006	0.994 (0.959–1.030)	0.749
8-ft up-and-go test	−0.025	0.975 (0.923–1.029)	0.360

Notes: with the exception of gender and education, all the independent variables are continuous

*Abbreviations*: *BMI* Body Mass Index, *MVPA* moderate-vigorous physical activity

^*^ *p* < 0.05

^**^ *p* < 0.001

## Appendix 6

**Table 13:** Variables that reached statistical significance on univariate logistic regression models according to the MEC-35, RAVLT-AT, QoL-AD and Goldberg depression scale

		B	EXP(B) (95% CI)	*p*-value
MEC-35	Chair sit and reach test	0.053	1.055 (1.011–1.100)	0.014
RAVLT-AT	Chair stand test	0.133	1.142 (1.030–1.266)	0.012
	Arm curl test	0.148	1.159 (1.041–1.291)	0.007
	Chair sit-and-reach test	0.053	1.054 (1.009–1.101)	0.018
QoL-AD	Steps/day	0.001	1.001 (1.000–1.001)	0.039
	Light physical activity	0.010	1.010 (1.002–1.019)	0.019
	Handgrip	0.061	1.063 (1.005–1.125)	0.034
	Chair stand test	0.110	1.116 (1.012–1.231)	0.028
	Arm curl test	0.166	1.181 (1.059–1.317)	0.003
Goldberg Depression Scale	Gender (Female)	−1.498	0.224 (0.062–0.812)	0.023
	Steps/day	0.002	1.002 (1.000–1.003)	0.008
	Light physical activity	0.014	1.014 (1.003–1.026)	0.014
	Arm curl test	0.177	1.194 (1.052–1.356)	0.006

Notes: with the exception of gender, all the independent variables are continuous

## Abbreviations

6MWT: 6 Minutes walking test
LT: Long term
MEC-35: Mini Examen Cognoscitivo-35
MVPA: Moderate to vigorous physical activity
QoL: Quality of life
QoL-AD: Quality of life-Alzheimer’s disease
RAVLT: Rey Auditory-Verbal Learning Test
SFT: Senior Fitness Test
